# Learning by aggregating experts and filtering novices: a solution to crowdsourcing problems in bioinformatics

**DOI:** 10.1186/1471-2105-14-S12-S5

**Published:** 2013-09-24

**Authors:** Ping Zhang, Weidan Cao, Zoran Obradovic

**Affiliations:** 1 Healthcare Analytics Research, IBM T.J. Watson Research Center, Yorktown Heights, NY 10598, USA; 2 School of Media and Communication, Temple University, Philadelphia, PA 19122, USA; 3 Center for Data Analytics and Biomedical Informatics, Temple University, Philadelphia, PA 19122, USA

## Abstract

**Background:**

In many biomedical applications, there is a need for developing classification models based on noisy annotations. Recently, various methods addressed this scenario by relaying on unreliable annotations obtained from multiple sources.

**Results:**

We proposed a probabilistic classification algorithm based on labels obtained by multiple noisy annotators. The new algorithm is capable of eliminating annotations provided by novice labellers and of providing a more accurate estimate of the ground truth by consensus labelling according to higher quality annotations. The approach is evaluated on text classification and prediction of protein disorder. Our study suggests that the higher levels of accuracy, effectiveness and performance can be achieved by the new method as compared to alternatives.

**Conclusions:**

The proposed method is applicable for meta-learning from multiple existing classification models and noisy annotations obtained by humans. It is particularly beneficial when many annotations are obtained by novice labellers. In addition, the proposed method can provide further characterization of each annotator that can help in developing more accurate classifiers by identifying the most competent annotators for each data instance.

## Background

In recent years, various groups studied the problem of developing classification models based on examples annotated by multiple labellers. The labels we integrate come from not only human beings (e.g., data curation tasks in modern biology, and crowdsourcing services) but also machine-based classifiers (e.g., protein disorder predictors).

From the methodology perspective of the multi-annotator problem, one line of research focuses on annotator filtering by identifying and excluding low-performing annotators [1-3]. The other line of research aims at a single consensus label by aggregating labels from multiple annotators [4-18]. Both strategies demonstrate significantly improved performance against single-annotator strategy and majority voting baselines.

Learning from multiple annotators is also applied to bioinformatics. For example, manually labelled data is successfully used together with mathematical models to provide annotator-specific accuracy estimates based on multi-annotator agreement [19,20]. In computer-aided diagnosis (CAD), many computer-aided image diagnosis systems [5,21-24] were built from labels (i.e., diagnoses) assigned by multiple physicians who provide their estimations of the gold standard, which can only be obtained from dangerous surgical operations. Also, Valizadegan et al. [25] developed a probabilistic approach for learning classification models from opinions provided by multiple doctors and applied the approach to Heparin Induced Thrombocytopenia (HIT) electronic health records (EHR). In the prediction of protein disorder, meta-learning is commonly used (e.g., metaPrDos [26], MD [27], PONDR-FIT [28], MFDp [29], MetaDisorder [30], and disCoP [31]). Meta predictors are typically developed relying on disorder/order labelled training datasets. These datasets contain a very small number of proteins which have not already been used for development of the component predictors. In addition, there is a potential problem of over-optimization for the meta predictors when combining information from multiple components. In contrast, here a meta predictor is constructed in a completely unsupervised process without use of confirmed disorder/order annotations [32].

In this study, we learn a classification model using multiple noisy labels obtained by multiple annotators. Specifically, we address a scenario where novice annotators are dominant. Our method for integration of multiple annotators by **A**ggregating **E**xperts and **F**iltering **N**ovices will be called AEFN. Based solely on the information obtained from the good annotators, in an iterative process our method evaluates annotators to exclude low-quality ones followed by re-estimation of the labels. In a scenario considered in our study the noisy annotations are obtained by a combination of humans and existing classification models. Therefore, the new method is applicable to many biomedical problems.

Compared to previous studies, the uniqueness of our study lies in the following aspects:

• The AEFN algorithm combines the removal of some annotators with labelling based on consensus of the remaining annotations. This is achieved without using any ground truth information.

• It provides estimates of good annotators' accuracy in addition to removing novice annotators.

• It is applicable in situations where annotators' accuracy varies across the data subsets which are not the case with previously proposed solutions (other than [9] and [10]).

• Compared to our previous study [33], AEFN algorithm is explored in more details by conducting additional experiments on prediction of protein disorder on CASP9 (i.e., the 9th Biannual Community Wide Experiment on the Critical Assessment of Techniques for Protein Structure Prediction held in year 2010) data. The new experiments with machine-based classifiers provide a complementary characterization to experiments on human annotators reported at the preliminary version [33]. In our solution, a combination of noisy annotations obtained by humans and existing machine-based classification models were integrated. Therefore, AEFN has the potential to be applied as a solution to many biomedicine and bioinformatics problems.

• Based on AEFN algorithm, a way of deciding which annotator is more appropriate to label new instances has been investigated in our experiments. This is potentially beneficial in any situation where annotating instances is expensive.

## Methods

Given a dataset *D*={***x****_i_*, *y_i_^1^*, ..., *y_i_^R^*}, where ***x****_i _*is an instance, *y_i_^j^*∈{0,1} is ***x****_i_*'s corresponding binary label which is provided by the *j*-th annotator. For multi-annotator problem the task is to get an estimation of the unknown true label *y_i_*.

Majority Voting (MV), a commonly used approach for this problem, has a limitation that the aggregated label for an example is estimated locally by only estimating the labels assigned to that example and not considering the performance of the labels for other examples.

In order to solve that problem, [8] introduced an MAP-ML algorithm. As [8] proposed "MAP-ML algorithm models the accuracy of the annotator separately on the positive and negative instances. If the true label is one, the sensitivity (true positive rate) *α^j ^*for the *j*-th annotator is the probability that the annotator labels it as one: *α^j ^*=Pr[*y_i_^j ^*=1| *y_i _*=1]. On the other hand, if the true label is zero, the specificity (1-false positive rate) *β^j ^*is the probability that annotator labels it as zero: *β^j^*=Pr[*y_i_^j ^*=0| *y_i _*=0]. Then MAP-ML corrects the annotator biases by jointly estimating the annotator accuracy (i.e., *α^j ^*and *β^j^*) and the hidden true label." For details of MAP-ML, please refer to [8].

MAP-ML implicitly assumes that the performance of the annotators (i.e., *α^j ^*and *β^j^*) doesn't depend on the examples. To fix this problem, GMM-MAPML algorithm takes into account that the annotators are not only unreliable, but may also be inconsistently accurate depending on the data. As [10] mentioned "GMM-MAPML models the annotators to generate labels as follows: given an instance ***x****_i _*to label, the annotators find the Gaussian mixture component which most probably generates that instance. Then the annotators generate labels with their sensitivities and specificities at the most probable component." For details of GMM-MAPML, please refer to [10].

Our previous study [33] goes further. As [33] argued "Recent experiments show that in some cases, a consensus labelling of a few experts will achieve better performance [32]. To further characterize the behaviour of annotators, we define the ranking evaluation score as *S^j^*=|*α^j^*+*β^j ^*-1|. Random annotations result in *S^j ^*near zero, while perfect annotations correspond to *S^j^*=1. Based on the ranking evaluation score, we propose an AEFN algorithm by extending the GMM-MAPML. In each iteration, ML estimation measures annotators' performance at each mixture component (i.e., their sensitivity $\alpha_{k}^{j}$ and specificity $\beta_{k}^{j}$). Then, we add a step to filter the low-quality annotators at each Gaussian component according to the score (i.e., the ranking evaluation score of the *j*-th annotator at the *k*-th Gaussian component): if $S_{k}^{j}$ is smaller than a pruning threshold, we filter the *j*-th annotator from the pool of annotators at the *k*-th Gaussian component. Thus, we refit the MAP estimation with only the good annotators and get the updated probabilistic labels *z_i _*based on the Bayesian rule." The algorithm is summarized at Algorithm 1 while details are provided at a preliminary version of this study [33].

**Algorithm 1: **AEFN Algorithm

**Input: **Dataset $D={\left\{x_{i},y_{i}^{1},...,y_{i}^{R}\right\}}_{i=1}^{N}$ containing *N *instances. Each instance has binary labels $y_{i}^{j}\in \left\{0,1\right\}$ from *R *annotators.

1: Find the fittest *K*-mixture-component GMM for the instances, and get the corresponding GMM parameters and components responsibilities τ_ik _for each instance.

2: Initialize $\Lambda_{k}=\left\{1,\ldots ,R\right\}$ the sets of good annotators for each Gaussian component *k*=1,...,*K*.

3: Initialize $z_{i}=\left(1/R\right)\sum_{j=1}^{R}y_{i}^{j}$ based on a majority voting.

4: Initialize iteration indication iter←0.

5: **repeat**

6:      (ML estimation)

7:      $\forall j\in \Lambda_{k}$, update the sensitivity $\alpha_{k}^{j}$ and specificity $\beta_{k}^{j}$ as follows

$$\begin{array}{l} \alpha_{k}^{j}=\sum_{i=1}^{N}z_{ik}y_{i}^{j}/\sum_{i=1}^{N}z_{ik}\\ \beta_{k}^{j}=\sum_{i=1}^{N}\left(\tau_{ik}-z_{ik})(1-y_{i}^{j}\right)/\sum_{i=1}^{N}\left(\tau_{ik}-z_{ik}\right) \end{array}$$

8:      Update the prior probability *p_i _*as $\sigma \left(w^{T}x_{i}\right)$.

9:      (Low-quality annotators filtering)

10:      **if **iter>0 (check from the second iteration)

11:           **for all ***k*=1,...,*K *(all Gaussian components) **do**

12:                **for all **$j\in \Lambda_{k}$**do**

13:                     Update $S_{k}^{j}=|\alpha_{k}^{j}+\beta_{k}^{j}-1|$.

14:                     **if **$S_{k}^{j}<\xi$ (the pruning threshold) **then**

15:                          $\Lambda_{k}\leftarrow \Lambda_{k}-\left\{j\right\}$

16:                     **end if**

17:                **end for**

18:           **end for**

19:      **end if**

20:      (MAP estimation)

21:      ∀i=1,…,N restricted to the annotators in the set $\Lambda_{k}$ instead of integrating all *R *annotators, estimate *z_i _*as follows

$$z_{i}=\frac{a_{i}p_{i}}{a_{i}p_{i}+b_{i}\left(1-p_{i}\right)}$$

where

$$p_{i}=\text{Pr}\left[y_{i}=1|x_{i},w\right]=\sigma \left(w^{T}x_{i}\right)$$

$$a_{i}=\overset{R}{\underset{j=1}{\prod }}{\left[\alpha_{q}^{j}\right]}^{y_{i}^{j}}{\left[1-\alpha_{q}^{j}\right]}^{1-y_{i}^{j}}$$

$$b_{i}=\overset{R}{\underset{j=1}{\prod }}{\left[1-\beta_{q}^{j}\right]}^{y_{i}^{j}}{\left[\beta_{q}^{j}\right]}^{1-y_{i}^{j}}$$

$$q=\underset{k=1,...,K}{\text{arg}\text{max}}\left(\tau_{ik}\right)$$

22: iter←iter+1(update the number of iterations)

23: **until **change of *z_i _*between two successive iterations< ξ.

24: Estimate the hidden true label *y_i _*by applying a threshold  γ on *z_i_*. That is, *y_i_*=1 if *z_i_*> γ and *y_i_*=0 otherwise.

Output:

• Detected low-quality annotators of all Gaussian components in set $\left\{1,\ldots ,R\right\}-\Lambda_{k}$.

• Good quality annotators of all Gaussian components in $\Lambda_{k}$ with sensitivity $\alpha_{k}^{j}$ and specificity $\beta_{k}^{j}$, for $j\in \Lambda_{k}$, *k*=1,...,*K*.

• The probabilistic labels *z_i _*and the estimation of the hidden true label *y_i_*, ∀i=1,…,N.

All multi-annotator algorithms are unsupervised meaning that integration of noisy labels is achieved without using true labels. Following properties differentiate the proposed AEFN algorithm from alternative multi-annotator approaches (i.e., MV, MAP-ML, GMM-MAPML): (1) It integrates labels globally (considers the accuracies of annotators globally and automatically assigns greater weights to more accurate annotators); (2) It is data-dependent (applicable in situations where annotators' accuracy varies across the data subsets); and (3) It filters novice annotators (eliminates novice annotations and estimates the consensus ground truth based only on expert annotations of high quality). Also we summarize the properties of all multi-annotator algorithms in the Table 1.

**Table 1:** Properties of multi-annotator algorithms.

Algorithms	Unsupervised?	Integrate labels globally?	Data dependent?	Filter novice annotation?
MV	Y	N	N	N
MAP-ML	Y	Y	N	N
GMM-MAPML	Y	Y	Y	N
AEFN	Y	Y	Y	Y

The comparisons of properties of multi-annotator algorithms are shown. 'Y' denotes that the algorithm has the property; 'N' denotes that the algorithm doesn't have the property.

## Results

In this section, we intend to validate the proposed AEFN algorithm by doing experiments on a biomedical text classification task and a protein disorder prediction task. The protein disorder prediction experiment with machine-based classifiers provides a complementary characterization to the usage of human annotators reported in the biomedical text classification experiments.

### Biomedical text classification experiment

In the experiment, we used a 1,000-sentence scientific texts corpus from Rzhetsky et al. [19]. For details of data pre-processing and experimental settings, please refer to [33].

In the preliminary version of this study [33], we showed that our AEFN was slightly better than GMM-MAPML, while it significantly outperformed other competitors, when all annotations were from experts. Using the same settings, our AEFN also selected a three-component GMM model with covariance matrix $\lambda D_{k}AD_{k}^{T}$for the biomedical text data. Shown in Table 2 and Table 3 are the filtered annotators and estimated sensitivity and specificity of each good annotator on the Evidence classification task and Focus classification task for each component. For the Evidence classification task, Annotator 1 has been filtered in the 1st and 3rd components, and Annotator 4 has been filtered in the 2nd component. For the Focus classification task, Annotator 5 has been filtered in all three components and Annotator 3 has been filtered in the 2nd and 3rd components. The tables show that for different tasks the annotators perform in different manners. For example, Annotator 5 is good at the Evidence classification task, but not at the Focus classification task. In addition, we found that the five annotators had comparable overall quality, and on average only one per component was eliminated. These results are consistent with the results of our preliminary version of this study [33].

**Table 2:** AEFN based accuracy estimates on the text evidence classification task without using ground truth.

	First Component		Second Component		Third Component	
Annotators	Estimated Sensitivity	Estimated Specificity	Estimated Sensitivity	Estimated Specificity	Estimated Sensitivity	Estimated Specificity
Annotator 1	Filtered		0.7573	0.7737	Filtered	
Annotator 2	0.8400	0.8445	0.8901	0.9303	0.8103	0.8798
Annotator 3	0.8984	0.9061	0.8150	0.8870	0.8235	0.8196
Annotator 4	0.7492	0.7553	Filtered		0.7184	0.8197
Annotator 5	0.8035	0.7810	0.7991	0.8199	0.8819	0.9152

The estimates by five annotators for three principal components on the text *evidence *task are shown.

**Table 3:** AEFN based accuracy estimates on the text focus classification task without using ground truth.

	First Component		Second Component		Third Component	
Annotators	Estimated Sensitivity	Estimated Specificity	Estimated Sensitivity	Estimated Specificity	Estimated Sensitivity	Estimated Specificity
Annotator 1	0.7672	0.7749	0.8005	0.7969	0.7634	0.7907
Annotator 2	0.9373	0.8588	0.8753	0.8271	0.8958	0.8863
Annotator 3	0.7383	0.8258	Filtered		Filtered	
Annotator 4	0.8059	0.8652	0.9010	0.8594	0.8318	0.8413
Annotator 5	Filtered		Filtered		Filtered	

The estimates by five annotators for three principal components on the text *focus *task are shown.

In [33], we also showed that our AEFN has much better AUCs than all competitor methods, especially when low-quality annotators dominate (e.g., 90% low-quality annotators and only 10% experts). To further characterize our AEFN method on annotator-performance estimation, we designed another experiment on the same biomedical text data as follows: (1) Find the fittest K-mixture-component GMM for all instances by using step 1 of AEFN. As discussed in the previous paragraph, we found a three-Gaussian-component model for the text data. (2) Randomly split 40% of instances as training data and the remaining 60% as testing data. (3) On training data, estimate annotators' performance and identify the best annotator for each Gaussian component by using our AEFN method. Here, we used the estimated ranking evaluation score as the criterion (the higher the better) to choose the best annotator. For the Evidence classification task, Annotator 3 was the best for the first component, Annotator 2 was the best for the second component, and Annotator 5 was the best for the third component. For the Focus classification task, Annotator 2 was the best for both the first and the third components, and Annotator 4 was the best for the second component. (4) On testing data, we compare three logistic regression classifiers: a) **Randomly Selected Annotator **that for each training data point used a label obtained by a randomly picked annotator among the five available annotators; b) **AEFN Indicated Annotator **that for each training data point picked an annotator based on the suggestion from (3); c) **Ground Truth **that is trained using an approximation of ground truth labels defined by the majority vote of the eight annotators' labels as previously discussed. The accuracies of these classifiers were compared according to 5-fold cross-validation on the 60% testing data. The purpose of using the 40% training data is to obtain annotator suggestion for AEFN Indicated Annotator classifier.

**Figure 1:**
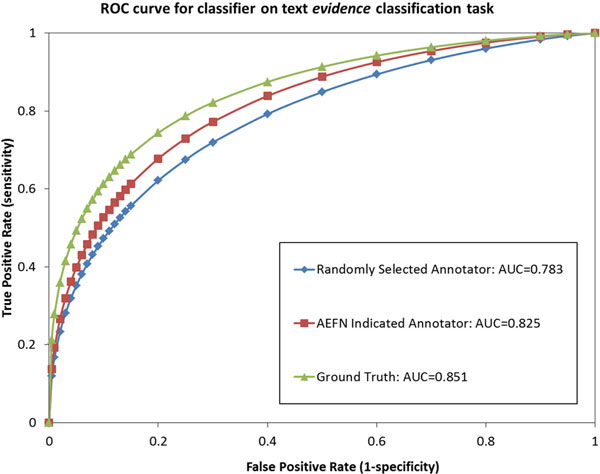
**Three logistic regression classifier ROC comparisons on the text evidence classification task**. The ROC comparison on the biomedical *evidence *classification of three strategies for selecting an annotation source for logistic regression. Methods are sorted in the legend of the figure according to their AUC values.

**Figure 2:**
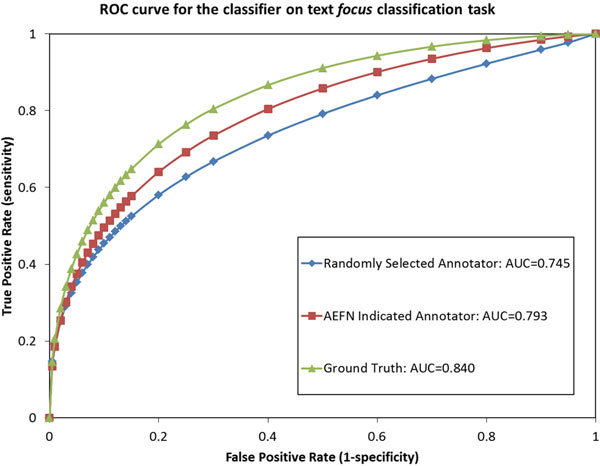
**Three logistic regression classifier ROC comparisons on the text focus classification task**. The ROC comparison on the biomedical *focus *classification of three strategies for selecting an annotation source for logistic regression. Methods are sorted in the legend of the figure according to their AUC values.

The ROC comparisons for three logistic regression classifiers on the Evidence and Focus classification tasks are shown in Figure 1 and 2, respectively. The figures show that when using the annotator's labels suggested by our AEFN method, a simple logistic regression method clearly outperforms the classifier trained using labels chosen randomly from five available annotators. The results show that our AEFN method can rank annotators by instance, and can help decide which annotator is more appropriate to label new instances. This is an interesting and important potential in the situation where annotating instances is expensive.

### Protein disorder prediction experiment

Treating an individual predictor as an annotator, the multi-annotator methods can be used to build meta-predictors for protein disorder prediction. In this section we experimentally validate the proposed algorithm on the CASP9 protein disorder prediction task. CASP9 data [34] consists of 117 experimentally characterized protein sequences with 2,427 disordered and 23,656 ordered residues. To reduce prediction noise due to experimental uncertainty, we didn't consider disorder segments shorter than four residues in the evaluation process. We selected 15 predictors developed by groups at different institutions, assuming that their errors are independent. Therefore we can treat them as individual annotators.

In the study, a feature vector (20 dimensions) of each residue was derived from the subsequence covered by a moving window centred at the current position. Of the 20 dimensions, the first 19 features come from amino acid frequencies composition and the last one is a local sequence complexity feature (based on the observation that low complexity regions are more likely to be disordered than ordered). For details of amino acid feature vector construction, please refer to [35]. In this experiment, we set the size of the moving window as 21, which is based on our previous study [32] as well as the ratio of short (<30 residues) disordered segments to long ones in the data.

Comparisons of 15 protein disorder predictors, the MV algorithm, the MAP-ML algorithm, the GMM-MAPML algorithm, and our AEFN algorithm on CASP9 data are shown in Table 4. Methods were evaluated by two measures [36]: the average of sensitivity and specificity (ACC), and the area under the ROC curve (AUC). Our proposed AEFN algorithm significantly outperforms the three competitor multiple-annotator methods (i.e., GMM-MAPML, MAP-ML, and MV) and each individual protein disorder predictor based on both ACC and AUC scores.

**Table 4:** CASP9 comparison on labelled data.

Predictor Name	Institution	ACC	AUC
AEFN		**0.801**	**0.887**
GMM-MAPML		0.785	0.874
MAP-ML		0.764	0.859
MV		0.735	0.776
PRDOS2	Tokyo Tech	0.754	0.855
MULTICOM-REFINE	U of Missouri	0.750	0.822
BIOMINE_DR_PDB	U of Alberta	0.741	0.821
GSMETADISORDERMD	IIMCB in Warsaw	0.738	0.816
MASON	George Mason U	0.736	0.743
ZHOU-SPINE-D	Indiana University	0.731	0.832
DISTILL-PUNCH1	UCD Dublin	0.726	0.800
OND-CRF	Umea University	0.706	0.759
UNITED3D	Kitasato University	0.704	0.780
CBRC_POODLE	CBRC	0.694	0.830
MCGUFFIN	University of Reading	0.688	0.817
ISUNSTRUCT	IPR RAS	0.676	0.739
DISOPRED3C	UCL	0.670	0.853
ULG-GIGA	University of Liege	0.588	0.726
MEDOR	Aix-Marseille U	0.579	0.679

Comparisons of AEFN vs. alternative multi-annotator methods (GMM-MAPML, MAP-ML and MV) and individual CASP9 protein disorder predictors.

**Figure 3:**
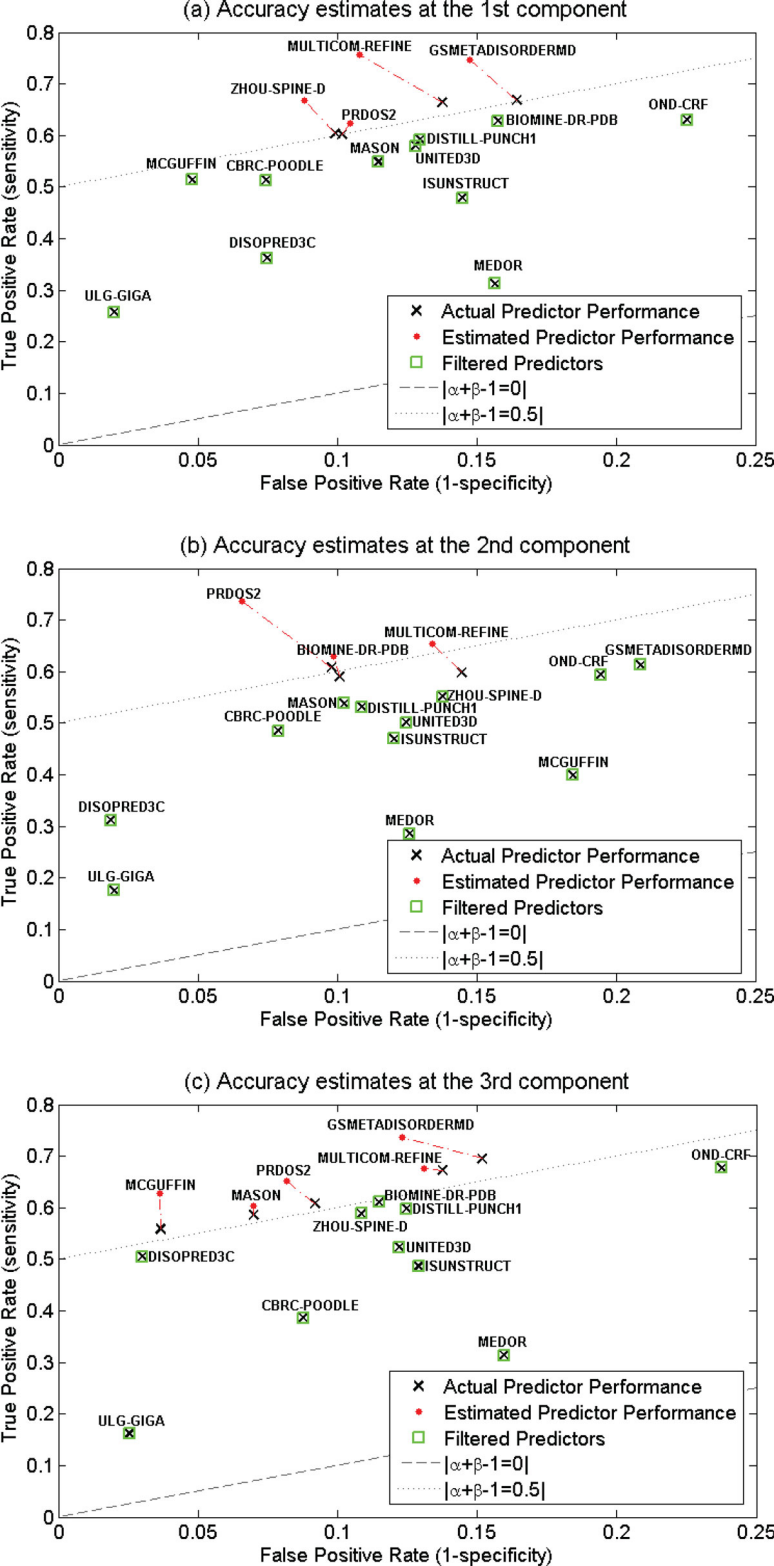
**Analysis of CASP9 disorder predictors at three components identified by AEFN**. In panels a, b, and c: the black cross plots the actual sensitivity and specificity of each predictor; the red dot plots the sensitivity and specificity of the best predictors as estimated by the AEFN algorithm; the green squares show the predictors filtered as those less accurate in the experiment.

For CASP9 data, AEFN algorithm also finds that a three-component GMM with the covariance matrix $\lambda_{k}$**B**_k_. For each component, estimated sensitivity and specificity of the best predictors, as well as filtered less-accurate predictors using AEFN, are shown in Figure 3. For comparison, we also plot the actual sensitivity and specificity of each individual predictor at each Gaussian component on the same figure. Figure 3 clearly shows that the individual CASP9 disorder predictors perform differently at different components. For example, GSMETADISORDERMD performs well in the first and third components, but it is not among the best in the second component. BIOMINE-DR-PDB performs well in the second component, but it is not among the best in the first and third components. The figure also demonstrates the main benefit of our proposed AEFN algorithm: the predictors identified as experts without relying on ground truth were indeed among the best according to their actual prediction performance at each component as verified by labelled data of confirmed order/disorder residues.

For further analysis, we found that the first, the second, and the third Gaussian components highly correlate with N-terminus (defined as 20% of residues at the start of a protein sequence), internal, and C-terminus (defined as 20% of residues at the end of a protein sequence) of protein sequences respectively. For details of the CASP9 amino-acid position distribution analysis, please refer to [10]. Based on the CASP9 analysis summarized in Figure 3 and the position distribution analysis, the only reliable predictors for all three regions are PRDOS2 and MULTICOM-REFINE (they are also the best individual predictors in the evaluation shown in Table 4). For N-terminal, reliable predictors also include ZHOU-SPINE-D and GSMETADISORDERMD while for the internal region we may also rely on BIOMINE_DR_PDB and for C-terminus we may also use MCGUFFIN, MASON, and GSMETADISORDERMD. The experiment provides evidence that AEFN algorithm can potentially be used to provide helpful suggestions on choosing the suitable disorder predictors for each region (N-terminus, internal, or C-terminus) of unknown protein sequences.

## Conclusions

A probabilistic algorithm (i.e., AEFN) for the multi-annotator classification problem is addressed in our study. Without using any ground truth information, the proposed AEFN algorithm is excluding lower quality annotations of novice labellers and providing more accurate classifications based on consensus of remaining experts' annotations of higher quality. Evaluation on biomedical text classification and prediction of protein disorder provides the evidence of the effectiveness of the proposed method. In our experiments, AEFN significantly outperformed alternatives that include the MV and multi-annotator algorithms (GMM-MAPML and MAP-ML). It was particularly beneficial when low-quality annotators are dominant. We have also found that AEFN algorithm can be used to determine which annotator is appropriate to label new instances. This is potentially beneficial in any situation where annotating instances is expensive. In addition, AEFN can be used for developing more accurate patient-specific diagnostic models by identifying groups of competent annotators for specific instances.

## List of abbreviations used

EHR: Electronic Health Record; MV: Majority Voting; ROC: Receiver Operating Characteristic; CASP: Critical Assessment of Techniques for Protein Structure Prediction.

## Competing interests

The authors declare that they have no competing interests.

## Authors' contributions

PZ conceived the algorithm, developed theoretical contributions, developed the proposed algorithm's prototype, performed experiments, carried out the analysis, and drafted the manuscript. WC reviewed theoretical contributions, and helped to revise the manuscript. ZO inspired the overall work, provided advice, and revised the final manuscript. All authors read and approved the final manuscript.

## Declarations

The publication costs for this article were funded by the corresponding author (ZO).

This article has been published as part of BMC Bioinformatics Volume 14 Supplement 12, 2013: Selected articles from the IEEE International Conference on Bioinformatics and Biomedicine 2012: Bioinformatics. The full contents of the supplement are available online at http://www.biomedcentral.com/bmcbioinformatics/supplements/14/S12.
